# Comparison of Toric and Spherical Orthokeratology Lenses in Patients with Astigmatism

**DOI:** 10.1155/2019/4275269

**Published:** 2019-02-20

**Authors:** Jun Jiang, Lili Lian, Feifu Wang, Ling Zhou, Xuhong Zhang, E. Song

**Affiliations:** ^1^Department of Ophthalmology, The Second Affiliated Hospital of Soochow University, Suzhou 215004, China; ^2^The Affiliate Eye Hospital of Wenzhou Medical University, Wenzhou 325027, China; ^3^Lixiang Eye Hospital of Soochow University, Suzhou 215021, China

## Abstract

**Purpose:**

This retrospective study aimed at comparing the efficacy and safety of toric and spherical orthokeratology lenses in the treatment of patients with moderate to high astigmatism.

**Methods:**

Fifty adolescents with myopia and moderate to high astigmatism (≥1.50 D) who underwent consecutive orthokeratology treatment for at least 1 year were included in this study. The toric group comprised 25 subjects (25 eyes, 11 M, 14 F; age, 10.67 ± 1.46 years) who were fitted with toric orthokeratology lenses. The spherical group comprised 25 subjects (25 subjects, 11 M, 14 F; age, 11.45 ± 1.63 years) who were fitted with traditional spherical orthokeratology lenses as a control. Corneal topography, visual acuity, axial length, and slit-lamp examinations were performed to determine the differences between these two groups. The corneal tangential difference mapping was conducted between baseline and every subsequent visit to calculate the magnitude of lens decentration. The corrective effect of ortho-K lens was measured by using the corneal axial difference map.

**Results:**

The mean decentration and its vertical vector were significantly less in the toric group than in the spherical group after 1 month of lens wear. In toric group, the corneal astigmatism decreased from 1.85 ± 0.31 D at baseline to 1.45 ± 0.85 D after the first month of wear. There was a significant linear correlation between the change in corneal astigmatism and lens decentration in the toric group from 1 month to 1 year (*Y* = 3.268 ∗ *X *+* *0.9182, *R*^2^ = 0.5035, *p* < 0.0001 (*X*: lens decentration; *Y*: astigmatic changes)). There were no significant differences in the post-OK uncorrected visual acuity, myopia control, or ocular health between the toric and spherical groups.

**Conclusion:**

The toric orthokeratology lens design can effectively reduce the lens decentration magnitude and CJ180 from 1-month visit to 12-month visit of patients with high or moderate corneal astigmatism. Meanwhile, there was no significant difference in visual acuity, myopia control, and ocular health throughout 12 months. However, the effect of toric lenses on corneal morphology may be susceptible to lens positioning.

## 1. Introduction

Myopia is considered one of the most common ophthalmological diseases and is associated with blurry distant vision and axial elongation [1, 2]. Myopia has become a global eye health problem. It is estimated that, in 2020, one-third of the worldʼs population (approximately 2.5 billion people) will be myopic [3]. Spectacles, contact lenses, and myopic refractive surgery are three effective methods for correcting myopia. Orthokeratology consists of the application of reverse geometry, rigid contact lenses as a nonsurgical treatment for myopia and has been widely used in recent years. Myopia is temporarily corrected through the night wear of lenses that flatten the front surface of the cornea to lessen the overall refractive power of the eye [4, 5]. Another important potential function of orthokeratology is effective control over the progression of myopia [4, 6–9]. Several studies have investigated the efficacy and magnitude of orthokeratology in refractive error correction and myopia control [4–9].

In addition to myopia, a high prevalence of astigmatism has also been reported in juveniles with myopia. Lisa et al. [10] reported that the prevalence of refractive and corneal astigmatism (≥1 D) in white school children in Northern Ireland was 20–24% and 25–29%, respectively. In the United States, children with astigmatism ≥2.00 D account for more than 20% of the total population [11, 12]. The incidence of astigmatism ≥1.00 D in Asia is approximately 10–20% [13, 14]. Moderate to high astigmatism is known as a relative contraindication to traditional orthokeratology lens fitting because of remarkable lens decentration and poor visual quality. Vinod et al. [15] have shown that greater degrees of corneal astigmatism are predicted to result in greater degrees of lens decentration. Different from the availability of myopia correction, orthokeratology lenses can only correct approximately 50% of corneal astigmatism [16]. Thus, high proportion of corneal astigmatism patients wearing orthokeratology lenses are likely to exhibit excessive residual astigmatism.

Toric orthokeratology lenses are specially designed orthokeratology lenses that adopt a spherical design in the optical zone but a toric design in the reverse curve and/or the alignment curve [17]. The lens and the cornea form a fit in the peripheral area and promote stable lens positioning. Pauné et al. [18] and Chen et al. [17] studied subjects with myopia > −5.50 D and astigmatism >1.25 D to investigate the effect of toric orthokeratology lenses. Similar results were obtained, with a significant reduction in the refractive power (Pauné: 106%; Chen: 81%), refractive astigmatism (Pauné: 85%; Chen: 79%), and corneal astigmatism (only in Chen's study: 44%). Toric orthokeratology lenses are effective for correcting low to moderate myopia with moderately high astigmatism.

In our understanding, toric lenses are considered a desirable choice for correcting myopia with moderate to high astigmatism. However, even if the centric position is established in the outset, the phenomenon of gradual lens decentration is occasionally observed in toric lens wearers in subsequent visits.

Orthokeratology modifies the cornea by its back surface [5]. Diverse back surface designs are likely to play different roles in the progression of corneal reshaping and myopia control. However, there are no published papers comparing the associated corneal changes and myopia progression of the toric design lens and spherical lens.

The primary purpose of this study was to compare the efficacy and safety of the two orthokeratology lens designs in the correction of patients with myopia and moderate to high corneal astigmatism over one year. The results of the study may help in predicting the corrective effects in patients with astigmatism and provide theoretical support for lens selection.

## 2. Methods and Subjects

### 2.1. Methods

This was a case-control study including all adolescents who had been fitted for orthokeratology lenses at the Eye Hospital of Wenzhou Medical University between 2014 and 2016. Based on the one-to-one match principle (same age, gender, proximate spherical equivalence, and corneal astigmatism), 25 eyes of 25 subjects were enrolled and included in the toric group and another 25 eyes of 25 subjects were enrolled in the spherical group. A series of regular ocular examinations (uncorrected visual acuity (UCVA), subjective refraction, corneal topography, intraocular pressure and tear break-up time, and axial length) were conducted before fitting the patient with trial lenses. Lens parameter selection was performed by the same experienced clinician. The first orthokeratology trial lenses were determined by the Sim K, and eccentricity values along the flattest meridian were calculated from the Medmont E300 corneal topographer (Medmont Studio 6 software version; Medmont International Pty, Ltd., Victoria, Australia). After a 20-minute trial in the clinic, the fluorescein pattern was evaluated to assess the suitability of the fit. The desired lenses were ordered based on the horizontal visible iris diameter, the fluorescein evaluation, and the over refraction result. Each patient was taught how to insert, remove, and care for the lens by professional clinicians in the hospital and instructed to wear the lens 8–10 hours per night. During subsequent routine visits, the subjects went to the clinic at 8–9 am while wearing the lenses, and the lenses were removed before their eyes were examined. The routine follow-up visits were scheduled at 1 day, 1 week, 1 month, 6 months, and 1 year. Each visit included UCVA measurements, corneal topography, and slit-lamp examinations. And, the axial length was recorded at the 1-year follow-up.

### 2.2. Subjects

In all, 50 adolescents were included in this study. They underwent orthokeratology treatment for at least one year and participated in regular follow-up visits. The subjects were divided into the toric and spherical lens design groups. Only one eye of each subject was included in this study. If both eyes met the inclusion criteria (Table 1), the right eye was chosen for analysis.

### 2.3. Orthokeratology Lens

The orthokeratology lenses used in this research were five-zone, reverse-geometry lenses (Lucid, Korea) consisting of Boston XO material (100 × 10^−11^(cm^2^·mlO^2^)/(s·ml·mmHg)). The lenses were designed with an overall diameter of 10.2–10.8 mm, a central optical zone diameter of 6 mm, and a central thickness of 0.23 mm. The toric lenses adopted a toric design for both the reverse and alignment curves. The lenses were designed according to the Jessen factor principle [19]: the myopia reduction was increased by 1 D per 0.2 mm, and the curvature radius of the orthokeratology lenses was flatter than the flat *K* value.

### 2.4. Measurements

#### 2.4.1. Visual Acuity

The monocular visual acuity of each subject was measured after they had picked up the lenses in the morning.

#### 2.4.2. Corneal Topography

Keratometry readings were measured using a Medmont E300 corneal topographer at baseline and every following visit. The corneal topographer is a placido disk-based video keratoscope that can calculate the axial curvature, the tangential curvature, and elevation data through a chord of 9 mm [20].

#### 2.4.3. Axial Length

The IOL-Master system (IOL-Master, Carl Zeiss, Germany) was used to measure the axial length of the eyeball before and after one year of orthokeratology lens use. Five continuous measurements were conducted, and the average data were automatically calculated for the record.

#### 2.4.4. Slit-Lamp Examination

The ocular health of each subject was evaluated by a specialist doctor. The evaluation included two aspects: an assessment of corneal staining and evaluations of some other ocular adverse reactions, such as corneal pressure and infiltration. For evaluating corneal staining, a fluorescein sodium strip wetted with 0.9% saline was used to touch the lower conjunctiva. The patient was told to blink until the fluorescein was evenly distributed on the corneal surface. The grading scale for corneal staining was as follows: grade 0, no significant corneal staining; grade I, slight scratches or slightly punctate stains; grade II, densely spotted corneal staining with mild discomfort; grade III, small areas of corneal epithelial defects with significant irritation; and grade IV: large areas of corneal epithelial defects with severe irritation.

### 2.5. Lens Decentration

**Figure 1:**
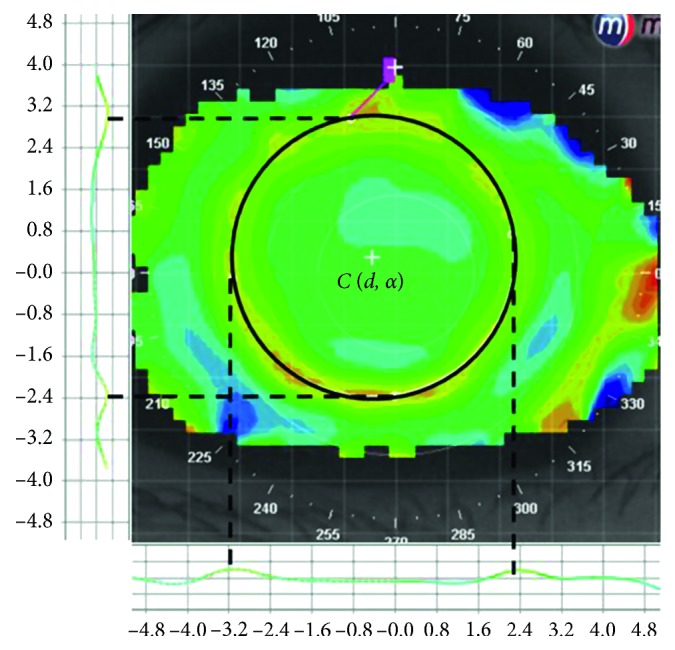
The lens positioning method used in the study.

Corneal tangential difference mapping was conducted between baseline and every visit during the orthokeratology treatment, and the results were analysed by two experienced, independent observers. In the reverse curve, the refractive variation resulting from the orthokeratology treatment showed a tendency to first increase and then decrease. Four maximum plots on the postorthokeratology astigmatism axis in this region were plotted to simulate an oval. The centre of the oval was defined as the centre of the lens treatment zone. The distance and angle between the apex of the cornea and the centre of the lens treatment zone were then measured, and the decentration was represented by horizontal and vertical vectors after vector decomposition for analysis. All decentered distances are recorded using absolute magnitudes. The accurate positioning method is shown in Figure 1.

### 2.6. Statistical Analysis

SPSS software (version 22.0; IBM, Armonk, NY, USA) was used for the statistical analysis of data in this study. The distribution of the data was analysed using the Kolmogorov–Smirnov normality test. Analysis of variance (ANOVA), Mann–Whitney *U* tests, and chi-squared tests were used to compare the average values between the groups. Repeated measures analysis of variance (RM-ANOVA) and post hoc *t* tests with Bonferroni corrections were used to assess changes in the corneal parameters during the follow-up period. The Greenhouse–Geisser correction was used to correct the experimental results if the significance level of Mauchly's sphericity test result was <0.05. The relations among the parameters involved in this research were analysed by Pearson correlation analysis. The intraclass correlation coefficient (ICC), coefficient of repeatability (COR), and standard deviation (SD) were used to evaluate the repeatability and reproducibility of the experiment, and *p* < 0.05 was considered statistically significant.

## 3. Results

### 3.1. Baseline Variables

A total of 50 subjects met the inclusion criteria and were included in this study (toric group: 25 subjects, 10.67 ± 1.46 years; spherical group: 25 subjects, 11.45 ± 1.63 years). There were no significant differences (*p* < 0.05) in the initial parameters between the two groups (*p* < 0.05) (Table 2).

### 3.2. Repeatability and Reproducibility

The repeatability and reproducibility of the localization method used in this study are shown in Table 3. The SD of three measurements by observers 1 and 2 was 0.030 ± 0.019 and 0.030 ± 0.016, respectively. The COR of the two observers was 7.53% and 8.19%, respectively, while the repeatability coefficient between the two observers was 7.02%. Cronbach's alpha coefficient and the ICC for each observer and between the observers were both greater than 0.95. Therefore, the use of such a locating method had acceptable repeatability and reproducibility.

### 3.3. Lens Decentration

**Figure 2:**
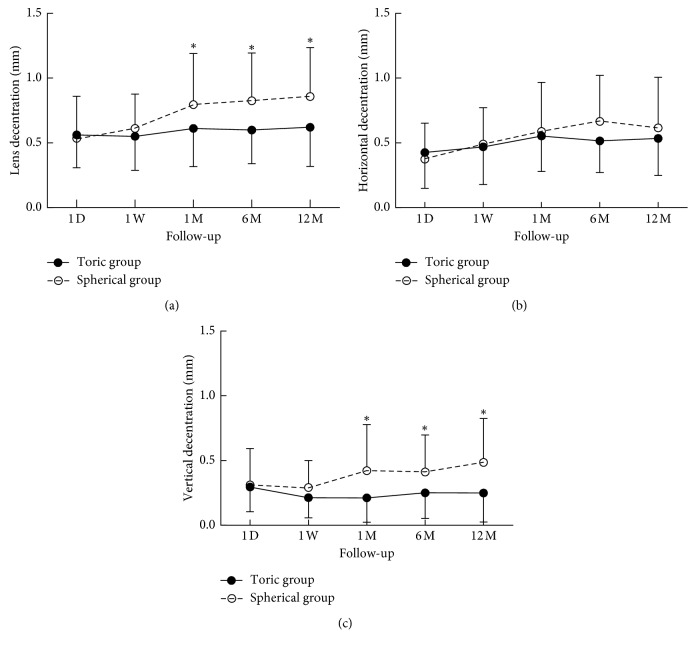
Orthokeratology lens decentration (a) and its horizontal (b) and vertical (c) vectors at all visits over 12 months. The upper and lower error bars represent the SDs for the spherical and toric groups, respectively. ^*∗*^indicates a statistically significant difference between the two groups.

The changes in lens decentration over time are presented in Figure 2. There were no significant changes in lens decentration or its decomposed vectors in the toric group over time (RM-ANOVA, *p* > 0.20) (Figure 2(a)). In the spherical group, lens decentration (Figure 2(a)) (post hoc, 1 month versus day 1, *p*=0.008) and its horizontal vector (Figure 2(b)) (post hoc, 1 month versus day 1, *p*=0.009) reached a significant increase by 1 month, with no further significant changes throughout the rest of the study period. While the vertical decentration vector in the spherical group increased significantly during the 1-year follow-up (RM-ANOVA, *F* = 2.909, *p*=0.025, Figure 2(c)), there were no other significant differences among the time points (post hoc, *p* > 0.05). The mean decentration and its vertical vector in the toric group were significantly less than those in the spherical group after 1 month of lens wear and at the later follow-ups (Figure 2(c)). No significant differences were found in the horizontal vector between the two groups (ANOVA, *p* > 0.05, Figure 2(b)).

### 3.4. Visual Acuity

**Figure 3:**
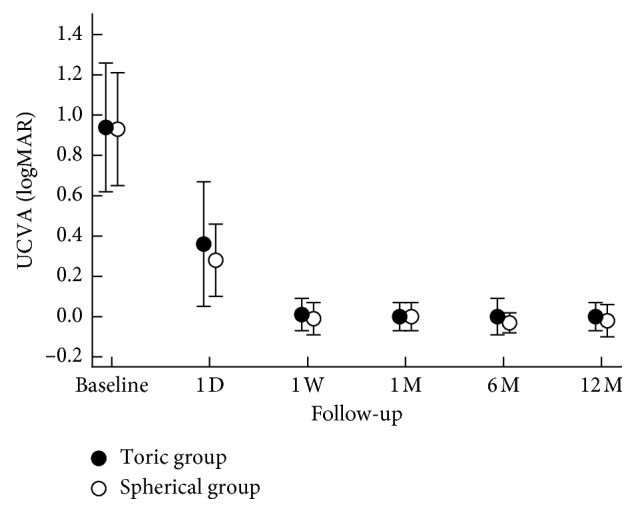
The uncorrected visual acuity (UCVA) for both groups at all visits over 12 months. The error bars represent the standard deviations of the mean value.

The UCVA showed significant improvement by the first day (toric group: 0.36 ± 0.31; spherical group: 0.28 ± 0.18) and appeared to stabilize by 1 week (toric group: 0.01 ± 0.08; spherical group: 0.01 ± 0.01 ± 0.08). There was no significant difference between the UCVA measured after one year of wear and the baseline best-corrected visual acuity (BCVA; ANOVA, *p* > 0.05). No significant difference was found in the UCVA between the two groups (ANOVA, *p* > 0.05). The above results could be seen in Figure 3.

### 3.5. Corneal Topography

The corneal equivalent power and apical power showed significant reductions in both groups over one year of orthokeratology lens wear (RM-ANOVA, *p* > 0.001). The changes reached statistical significance by 1 day and appeared to stabilize by 1 month.

**Figure 4:**
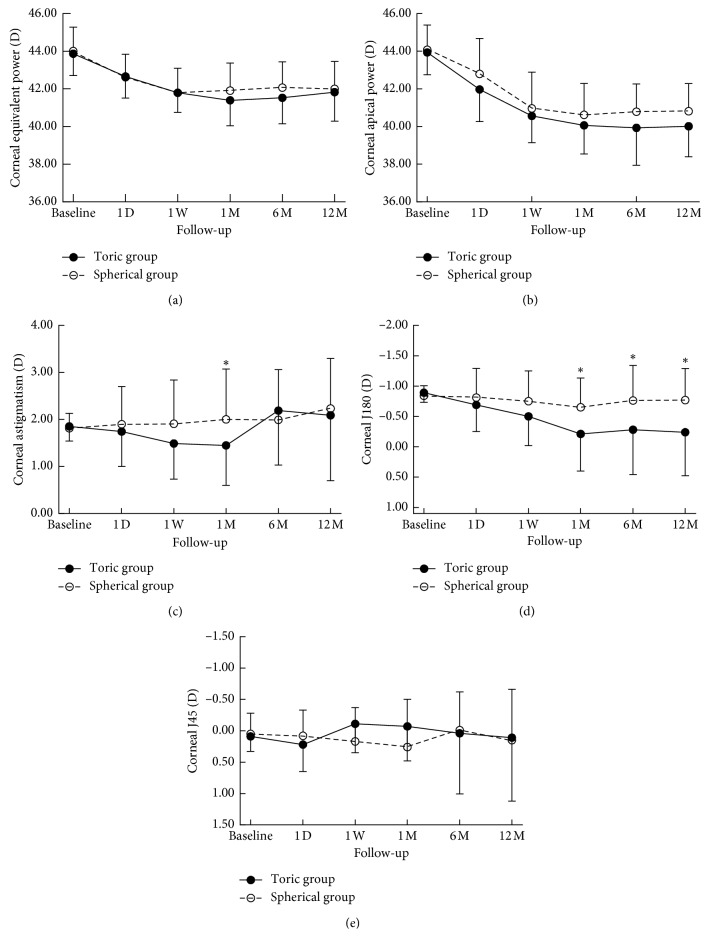
Change of corneal equivalent power (a), corneal apical power (b), corneal astigmatism (c), Corneal J180 (d), and Corneal J45 (e) at all visits over 12 months. The upper and lower error bars represent the standard deviations of the spherical and toric groups, respectively. ^*∗*^indicates a statistically significant difference between the two groups.

There were no significant differences between the two groups at any follow-up stage (ANOVA, *p* > 0.05). The above results could be seen in Figures 4(a)and 4(b).

The corneal astigmatism in the toric group showed a significant change over one year (RM-ANOVA, *p* > 0.020). The corneal astigmatism decreased from 1.85 ± 0.31 D at baseline to 1.45 ± 0.85 D after the first month of wear and increased to 2.19 ± 1.16 D by 6 months, subsequently decreasing to 2.09 ± 1.39 D by 1 year. There were no significant changes in the corneal astigmatism in the spherical lens group at any visit (RM-ANOVA, *p* > 0.234). Comparison of the corneal astigmatism between the two groups at each follow-up stage showed significantly less astigmatism in the toric group than in the spherical group only after 1 month of orthokeratology lens wear (ANOVA, *p* > 0.048). The above results could be seen in Figure 4(c).

The CJ180 in the toric group decreased significantly overall visits (RM-ANOVA, *p* > 0.001) and decreased by approximately 0.87 D from baseline over 1 year (95% confidence interval: 0.613–1.132 D, post hoc, *p* > 0.003). There were no significant changes in the CJ180 in the spherical group over 1 year (RM-ANOVA, *p* > 0.05). The CJ180 in the toric group was significantly less than that in the spherical group at 1 month, 6 months, and 1 year (ANOVA, *p* > 0.05). There were no significant changes in the corneal J45 component (CJ45) in the two groups over 1 year (RM-ANOVA, *p* > 0.05), and there were no statistically significant differences between the two groups (ANOVA, *p* > 0.05). The above results could be seen in Figures 4(d) and 4(e).

### 3.6. Myopia Reduction and Axial Length

**Figure 5:**
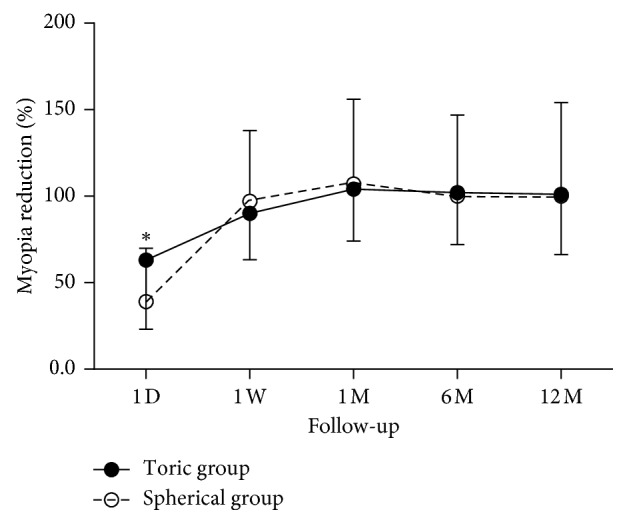
Myopia reduction in the two groups at all visits over 12 months. The upper and lower error bars represent the standard deviations of the spherical and toric groups, respectively. ^*∗*^indicates a statistically significant difference between the two groups.

**Figure 6:**
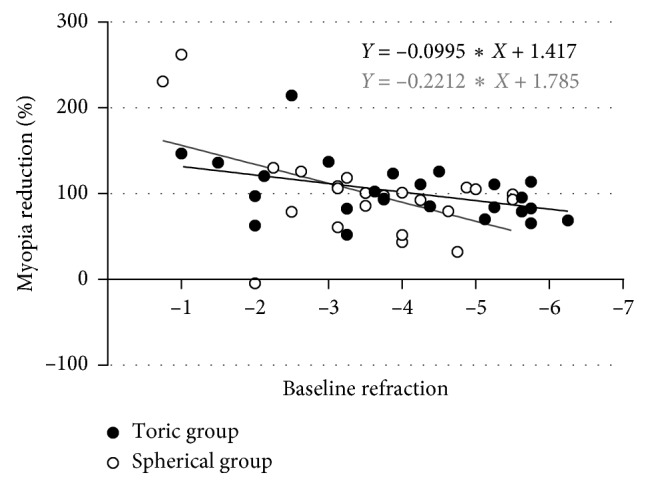
Linear correlation between the myopia reduction and the baseline refraction in the two groups.

The myopia reduction was expressed as a percentage of the initial spherical equivalent power in the change in the corneal apical power based on corneal axial difference map (e.g., initial refraction: −3.00/−1.00 × 180; initial spherical equivalent power: 3.00 + 1.00/2 = 3.50 D; corneal axial difference map : baseline vs 1 month = 3.00 D; myopia reduction = 3.00/3.50 = 85.71%) [21]. On the first day, the reduction in the toric group was 63 ± 0.44%, which was significantly greater than that in the spherical group, as 39 ± 0.31% (ANOVA, *p*=0.037, Figure 5). There was no significant difference between the two groups after 1 week, 1 month, 6 months, or 12 months of lens wear (ANOVA, *p* > 0.05, Figure 5). There was significant linear correction between the myopia reduction and baseline refraction (equivalent spherical power) in both groups (Figure 6). One-way ANOVA was performed to assess changes in the axial length over the 12-month period between the two groups of subjects. The results showed no significant differences in the axial length changes between the two groups (toric group: 0.13 ± 0.18; spherical group: 0.11 ± 0.20, *p* > 0.05).

### 3.7. Ocular Health

There were no serious complications, such as corneal infiltration and keratitis, during the study period in either group. Only grade I corneal staining was found in both groups over 1 year. The incidence of spotting in both groups was highest on the first day (toric group: 40%; spherical group: 32%), and there were no significant differences in the incidence of corneal staining between the two groups at various time points (chi-squared test, *p* > 0.05). All corneal staining could be effectively cured by the administration of artificial tears and antibiotic eye drops and not wearing the orthokeratology lenses for a few days. Corneal pressure traces were observed in 3 subjects in each group over 1 year.

## 4. Discussion

Central and stable lens positioning has always been a sign of successful orthokeratology lens fitting. There have been some previous studies involving the possible influencing factors of spherical orthokeratology lens decentration. Vinod et al. [15] noted that there was a positive correlation between corneal astigmatism and spherical lens decentration, and a negative correlation was found between the corneal curvature and lens decentration. A study by Li et al. [22] showed that the asymmetry of the cornea may be an important cause of lens decentration. In our experiment, there were no significant differences in the above corneal parameters between the two groups at baseline (*p* > 0.05). The difference in lens design might be the main factor affecting lens decentration.

In this study, the lens position showed different trends over time in the two groups. The lens position maintained a steady state in the toric group but showed an increasing trend to decentration in the first month in the spherical group. In other words, significantly less decentration was observed in the toric group than in the spherical group after 1 month of lens wear. The mean difference in the decentration and its horizontal and vertical vectors between the two groups at 1 year was 0.237 mm, 0.082 mm, and 0.236 mm, respectively. The reduced alignment between a spherical lens and toric cornea may be one explanation for this difference. Moreover, the first month is a critical period for corneal reshaping in orthokeratology [23]. Changes in the corneal morphology also lead to dynamic changes in the lens position within 1 month. Due to the toric design of the midperipheral zone, the position of toric lenses may be less affected by the gradual flattening of the central corneal curvature. The toric lenses showed less decentration mainly in the vertical direction, and the spherical lenses showed a tendency towards downward decentration. Previous studies by Vinod et al. [15] and Chen et al. [24] reported a similar result: inferotemporal decentration was most commonly observed in patients with astigmatism wearing spherical lenses. All patients included in this study had with-the-rule astigmatism; thus, the lens was more likely to move up and down with the eyelids. In addition, the effect of gravity on the lens might also be a factor of the above phenomenon.

Numerous previous studies have shown the changes in corneal topography for these two types of lenses at different time points separately. For traditional spherical orthokeratology lenses, most studies have shown that no extra astigmatism was caused in subjects without astigmatism, but the results in patients with astigmatism have been controversial. Cheung et al. [25] conducted a study of spherical lenses in patients with refractive astigmatism of ≤0.75 D for 6 months and found no significant changes in the corneal astigmatism or CJ180 and CJ45 components. Mountford [16] used the Bailey–Carney method combined with the Alpins method to find that spherical lenses can correct approximately 50% of corneal astigmatism. In the case of toric lenses, conclusions obtained from different studies have been consistent: toric orthokeratology lenses can partially correct corneal astigmatism. This study reflects the overall changes occurring over 1 year of wearing two types of lenses, which has not been previously investigated. Absence of significant changes in corneal astigmatism in the spherical group is consistent with the results obtained by Cheung et al. [25]. The toric group showed two trends over the course of 1 year (the corneal astigmatism decreased from 1.85 ± 0.31 D at baseline to 1.45 ± 0.85 D after the first month of wear and increased to 2.19 ± 1.16 D by 6 months). However, the corneal astigmatism and CJ180 decreased significantly within 1 month, which is consistent with the results obtained by Chen [17] and Pauné [18] in 1 month.

The two lens types showed different trends regarding changes in the corneal astigmatism over one year. The corneal astigmatism was significantly different between the two groups only after 1 month (*p*=0.026). The corneal astigmatism can be decomposed into the transverse or longitudinal component, CJ180, and the oblique component, CJ45. Only the CJ180 of the toric group showed a significant decreasing trend during the follow-up period and decreased significantly by 73% after 1 year of lens wear. A correlation analysis was performed for the changes in corneal astigmatism, CJ180 and CJ45. The change in corneal astigmatism in the toric group was significantly correlated with the change in CJ180 after 1 month (*R* = 0.666, *R*^2^ = 0.43). This correlation was lost after 6 months and 1 year. Moreover, correlation analysis between lens deviation and corneal astigmatism showed a significant linear correlation between the change in astigmatism and lens decentration from 1 month to 1 year. The linear regression equation was *Y* = 3.268 ∗ *X *+* *0.9182, *R*^2^ = 0.5035, *p* < 0.0001 (*X*: lens decentration; *Y*: astigmatic changes). The significant increase in corneal astigmatism after 1 month can be explained by the following slight decentration. There was no significant correlation between the above two variables in the spherical group.

In this study, the incidence of non-with-the-rule astigmatism was obtained at the different follow-up stages in the two groups, with 12%, 28%, 52% 48%, and 44% in the toric group and 12%, 16%, 16%, 16%, and 24% in the spherical group at 1 day, 1 week, 1 month, 6 month, and 1 year, respectively. The astigmatism in the axial direction in the toric group showed a significant change during lens wear, and the incidence of this change was significantly greater in the toric group than in the spherical group (chi-squared analysis, *p* < 0.05). The change in the astigmatic axis may account for the inconsistency between the corneal astigmatism and CJ180 changes in the toric group after 1 month. The toric lens and the astigmatic cornea form a 360° confined space in the peripheral zone, so the lens misalignment causes morphological changes in the cornea, resulting in additional astigmatism.

The apical corneal power showed a significant linear correlation with the subjective refractive result and was used to calculate the myopia reduction. Although there was no significant difference in visual acuity between the two groups, the toric group showed faster correction and a reduced possibility of temporary central corneal power increase on the first day (Figure 5). The toric orthokeratology lens appeared to show a better corrective effect than the spherical lens in the patients with moderate to high myopia (≤3.00 D) (Figure 5). For patients with high myopia and lower corneal astigmatism, toric orthokeratology lenses can also be actively considered.

Both groups showed acceptable visual acuity and myopia control after 1 year of lens wear. The axial length changes showed myopia control effects similar to those reported by Chen [9] and Pauline [8] et al. The most common adverse reaction in both groups was corneal staining, which is considered reversible and could be cured by short-term treatments. Our results suggest that both orthokeratology lens types can effectively correct myopia in adolescents with moderate to high astigmatism.

## 5. Conclusion

The toric orthokeratology lens design can effectively reduce the lens decentration magnitude and CJ180 from 1-month visit to 12-month visit of patients with high or moderate corneal astigmatism. Meanwhile, there was no significant difference in visual acuity, myopia control, and ocular health throughout 12 months. However, the effect of toric lenses on corneal morphology may be susceptible to lens positioning.

## Figures and Tables

**Table 1:** Inclusion and exclusion criteria.

*Inclusion Criteria*
1. 8 ≤ Age ≤15 at baseline
2. Best-corrected distance monocular visual acuity (BCVA) ≤0.00 logMAR
3. Spherical refractive error(noncycloplegic subjective refraction) ≥−6.00 D
4. With-the-rule corneal astigmatism(noncycloplegic subjective refraction) ≥1.50 D
5. Corneal flat *K* value 41.00 D∼46.00 D
6. No orthokeratology fitting contraindication
7. No history of ocular surgery and trauma
8. No history of other contact lens wearing
9. No current systemic or ocular conditions that may affect lens wear

*Exclusion Criteria*
1. Follow-up irregularly
2. Corneal topography defect 20% or above
3. Change lenses in the follow-up period

**Table 2:** Baseline parameters (mean ± SD) of the two groups.

Parameters	Toric lens group 25 patients, 25 eyes	Spherical lens group 25 patients, 25 eyes	*p* value
Age (y)	10.67 ± 1.46	11.45 ± 1.63	0.078
Gender (male/female)	11/14	11/14	1.000
Refractive M (D)	−4.01 ± 1.46	−3.64 ± 1.37	0.366
UCVA (logMAR)	0.94 ± 0.32	0.93 ± 0.28	0.866
BCVA (logMAR)	−0.04 ± 0.04	−0.03 ± 0.04	0.779
Axial length (mm)	25.12 ± 0.90	25.01 ± 0.81	0.645
Corneal equivalent power (D)	43.87 ± 1.16	44.01 ± 1.27	0.680
Corneal apical power (D)	43.94 ± 1.19	44.08 ± 1.32	0.696
Corneal toricity (D)	1.85 ± 0.31	1.81 ± 0.32	0.611
Corneal J180 (D)	−0.89 ± 0.16	−0.84 ± 0.17	0.286
Corneal J45 (D)	0.09 ± 0.24	0.05 ± 0.33	0.977

UCVA : uncorrected visual acuity; Corneal J180 = −C cos 2*θ*/2; BCVA: best-corrected visual acuity; Corneal J45 = −C sin 2*θ*/2 (C: corneal astigmatism power, *θ*: corneal astigmatism axis).

**Table 3:** Repeatability and reproducibility of orthokeratology lens decentration measurements.

		SD (mm)	COR (％)	Cronbach's alpha	ICC (95％ CI)
For each observer	Observer 1	0.030 ± 0.019	7.25	0.995	0.987 (0.979–0.992)
	Observer 2	0.030 ± 0.016	7.75	0.996	0.987 (0.979–0.992)
Between observers		0.024 ± 0.022	6.51	0.993	0.993 (0.989–0.996)

SD : standard deviation; COV : coefficient of repeatability; ICC : intraclass correlation coefficient.

## Data Availability

The data used to support the findings of this study are available from the first author upon request.
